# Role of long-chain acyl-CoAs in the regulation of mycolic acid biosynthesis in mycobacteria

**DOI:** 10.1098/rsob.170087

**Published:** 2017-07-19

**Authors:** Yi Ting Tsai, Valentina Salzman, Matías Cabruja, Gabriela Gago, Hugo Gramajo

**Affiliations:** Laboratory of Physiology and Genetics of Actinomycetes, Instituto de Biología Molecular y Celular de Rosario (IBR-CONICET), Facultad de Ciencias Bioquímicas y Farmacéuticas, Universidad Nacional de Rosario, Rosario, Argentina

**Keywords:** mycobacterium, mycolic acid, acyl-CoA, isoniazid, transcriptional regulation

## Abstract

One of the dominant features of the biology of *Mycobacterium tuberculosis*, and other mycobacteria, is the mycobacterial cell envelope with its exceptional complex composition. Mycolic acids are major and very specific components of the cell envelope and play a key role in its architecture and impermeability. Biosynthesis of mycolic acid (MA) precursors requires two types of fatty acid synthases, FAS I and FAS II, which should work in concert in order to keep lipid homeostasis tightly regulated. Both FAS systems are regulated at their transcriptional level by specific regulatory proteins. FasR regulates components of the FAS I system, whereas MabR and FadR regulate components of the FAS II system. In this article, by constructing a tight *mabR* conditional mutant in *Mycobacterium smegmatis* mc^2^155, we demonstrated that sub-physiological levels of MabR lead to a downregulation of the *fasII* genes, inferring that this protein is a transcriptional activator of the FAS II system. *In vivo* labelling experiments and lipidomic studies carried out in the wild-type and the *mabR* conditional mutant demonstrated that under conditions of reduced levels of MabR, there is a clear inhibition of biosynthesis of MAs, with a concomitant change in their relative composition, and of other MA-containing molecules. These studies also demonstrated a change in the phospholipid composition of the membrane of the mutant strain, with a significant increase of phosphatidylinositol. Gel shift assays carried out with MabR and P*fasII* as a probe in the presence of different chain-length acyl-CoAs strongly suggest that molecules longer than C_18_ can be sensed by MabR to modulate its affinity for the operator sequences that it recognizes, and in that way switch on or off the MabR-dependent promoter. Finally, we demonstrated the direct role of MabR in the upregulation of the *fasII* operon genes after isoniazid treatment.

## 1. Background

*Mycobacterium tuberculosis*, the causative agent of tuberculosis, owes it successful colonization and survival characteristics to its adaptation to the varying conditions imposed by the host tissue physiology and immune response [1]. This adaptation depends on the coordination of gene expression via the regulation of transcription, and in *M. tuberculosis* this is achieved by the collective action of a set of approximately 200 transcription factors and DNA-binding proteins and 13 different *σ* factors [2,3], which allow the bacterium to modify its expression profile in response to a given environment. Recently, a high-throughput approach employing ChIP-seq and transcriptional profiling was developed to identify the genes controlled by approximately 80% of transcription factors present in *M. tuberculosis* [4–6]. The results obtained suggested that the control of gene expression in this pathogen may involve a layer of complexity that is currently unappreciated.

*Mycobacterium tuberculosis*, as well as other related species of mycobacteria, has a distinctive cell envelope which plays a crucial role in the process of pathogenesis, virulence and survival by conferring on them a resistance cover to face adverse niches. The main components of this peculiar cell wall are the mycolic acids (MAs), which are found either attached to the arabinogalactans, that together with peptidoglycan form the cell wall skeleton [7,8], or unbound as free fatty acids (FAs) that contribute to the pathogen's persistence [9]. Biosynthesis of MA precursors requires the coordinated action of at least two different types of FA synthases (FASs): the eukaryotic-like multifunctional enzyme FAS I and the multienzyme complex FAS II, consisting of a series of discrete mono-functional proteins, each catalysing a reaction in the elongation pathway. Unlike the type II synthases of other bacteria, the mycobacterial FAS II is incapable of *de novo* FA synthesis from acetyl-CoA; instead, it elongates medium-chain-length C_12_–C_16_ fatty acyl-CoAs previously synthesized by FAS I [10] to generate very long-chain meromycolyl-ACPs. In mycobacteria, FAS I performs *de novo* biosynthesis of acyl-CoAs in a bimodal manner by generating C_12_–C_18_ and C_24_–C_26_ esters of CoA [11,12]. Recent studies carried out on a *Mycobacterium smegmatis* FAS I conditional knockdown mutant showed the essentiality of this enzyme for its survival, and surprisingly it was also found that even in the absence of FAS I, MA biosynthesis was unaffected, with triacylglyceride (TAG) degradation being the source of the substrates for FAS II elongation [13]. It is still unclear why the bacteria only continue with the production of MAs upon reduction of *de novo* FA biosynthesis, since these products are also the building blocks for phospholipids (PL) and TAG formation. Nevertheless, a clear upregulation of genes that encode for the FAS II system was observed, suggesting a crosstalk between the two FAS systems at the level of gene expression when lipid homeostasis is disrupted by a conditional FAS I depletion [13]. Therefore, we can infer that FAS I and FAS II systems should be strictly co-regulated in order to maintain lipid homeostasis in this microorganism.

The relevance of lipid metabolism for the survival of *M. tuberculosis* and the successful progression of infection are clearly highlighted by the number of first-line drugs used for tuberculosis treatment, such as isoniazid (INH), ethionamide (ETH) and thiolactomycin (TLM), all of them targeting the bacterial lipid metabolism. In contrast with the wealth of knowledge about the biogenesis of mycobacteria cell envelopes, little is known about the regulatory circuits that control these processes. Recent studies have started to shed light on how mycobacteria precisely control the cell wall biosynthesis, and they indicate that these organisms employ many sophisticated regulatory strategies such as transcriptional regulation of lipid genes or modulation of the activities of key enzymes by post-translational modification [14–16]. Therefore, by fine-tuning the levels and activities of biosynthetic enzymes, mycobacteria constantly match the demand for new membrane synthesis and extension of their complex cell wall.

It has been demonstrated that the transcriptional regulator MabR controls the expression of essential *fasII* operon genes involved in MA biosynthesis by binding palindromic inverted repeat sequences found in the P*fasII* promoter region [17]. A second transcription factor named FadR has also been suggested as a *fasII* regulator, although there is no conclusive evidence of such regulation *in vivo* [18]. More recently, FasR, a transcriptional activator of the *fas-acpS* operon, which encodes for the key enzymes FAS I and the phosphopantetheinyl transferase AcpS, was identified and characterized [19]. Furthermore, MabR and FasR have also been demonstrated to be essential in *M. smegmatis*, in contrast with most transcriptional regulators that regulate FA metabolism in other bacteria [17,19].

In the original characterization of MabR, a series of experiments carried out in conditions of *mabR* overexpression had suggested that this protein could function as a negative regulator of the *fasII* operon. However, further studies on this regulator and other general considerations led us to revisit the physiological role of MabR in mycobacteria. (i) A MabR orthologue found in *Streptomyces coelicolor*, named FasR, was found to induce the expression of the *fasII* operon genes in this organism. *Streptomyces coelicolor* FasR has 42% identity with MabR and recognizes highly similar inverted repeat sequences on the DNA, which constitutes the operator region recognized by this regulator in the *PfasII* promoter of *S. coelicolor* [20]. The difference between these two systems is that in *S. coelicolor* FAS II is responsible for adjusting the FA composition of the cell membranes and storage lipid compounds, while in mycobacteria the FAS II system is involved in the meromycolic acid biosynthesis. (ii) The location of the predicted FasR-binding site in the *PfasII* promoter region of *S. coelicolor*, around −67 bp from the transcriptional start site (TSS), is consistent with its function as an activator. Interestingly, MabR-binding sequences in *M. tuberculosis* and *M. smegmatis* are located at −87 and –147 from the TSS of the corresponding *PfasII* promoters, respectively, which correspond, in most cases, to operator regions recognized by activators [21]. (iii) The fact that MabR is an essential transcriptional regulator is also consistent with the idea of an activator and not with a repressor protein.

Most of the bacterial transcription factors involved in lipid biosynthesis exert their regulation by sensing effector molecules that are either substrates or final products (e.g. malonyl-CoA, long-chain acyl-CoAs or acyl-ACPs) of FA biosynthesis [22]. In mycobacteria, we found that the affinity of FasR for its DNA-binding sequences is negatively modulated by long-chain acyl-CoAs [19], while for FadR it was also shown that FadR–DNA binding can be impaired by long-chain fatty acyl-CoA compounds.

In this work, we present the characterization of a tight *mabR* conditional mutant in *M. smegmatis* which allowed us to demonstrate that MabR is a transcriptional activator of the *fasII* operon. We also carried out *in vitro* MabR–DNA binding studies and *in vivo PfasII–lacZ* transcriptional fusion experiments to identify the possible effector(s) or signal molecule(s) that modulate the activity of MabR. Finally, we investigated the role of MabR in the transcriptional upregulation of the *fasII* operon genes as a response to the lipid imbalance generated in INH-treated mycobacteria.

## 2. Material and methods

### 2.1. Bacterial strains, culture and transformation conditions

The *Escherichia coli* strain DH5α [23] was used for routine subcloning and was transformed according to Sambrook *et al.* [24]. The transformants were selected on media supplemented with the appropriate antibiotics. Strain BL21 (λDE3) is an *E. coli* B strain lysogenized with λDE3, a prophage that expresses the T7 RNA polymerase from the isopropyl-β-d-thiogalactopyranoside (IPTG)-inducible *lac*UV5 promoter [25]. *Mycobacterium smegmatis* mc^2^155 is an electroporation-efficient mutant of mc^2^6 [26]. Liquid cultures of *M. smegmatis mabR* cKD and WT-pMT13 were grown in 7H9 medium supplemented with 0.2% glycerol and 0.03% Tyloxapol, in the presence of 2.5, 25 or 200 ng ml^−1^ of anhydrotetracycline (ATc). Hygromycin (Hyg, 50 μg ml^−1^), apramycin (Am, 50 μg ml^−1^), kanamycin (Kan, 15 μg ml^−1^) and streptomycin (Str, 12 μg ml^−1^) were added when needed. All the cultures were incubated at 37°C with gentle shaking (180 r.p.m.). Recombinant plasmid and strain genotypes are listed in the electronic supplementary material, tables S1 and S2, respectively.

### 2.2. DNA manipulation, plasmid construction and mutant generation

Isolation of plasmid DNA, restriction enzyme digestion and agarose gel electrophoresis were carried out by conventional methods [24]. Genomic DNA of *M. smegmatis* was obtained as described [27].

#### 2.2.1. pMR10

*MSMEG_4324* (*mabR*_MS_) was amplified from genomic DNA of *M. smegmatis* mc^2^155 using the oligonucleotides N-MS4324 (5′-CATATGCCCGACAATCGGTTCGTTC-3′) and C-MS4324 (5′-GAATTCACTAGTGATCTGTGATCCGTACCT-3′) to introduce an *Nde*I site at the translational start codon of *mabR* gene and *EcoR*I and *Spe*I sites at the end of the ORF; the corresponding restriction sites are underlined. To generate an *mabR*_MS_ His tag fusion gene, the PCR product was digested with *Nde*I and *EcoR*I and cloned into *Nde*I/*EcoR*I-cleaved pET24a(+), yielding pMR10.

#### 2.2.2. pCK3

A synthetic DNA fragment containing P*_myc1_tetO* [28] and its terminator sequence was introduced in plasmid pUC57. The resulting plasmid was digested with *Kpn*I and *Hin*dIII, and the fragment was cloned into pMV306 previously digested with the same enzymes.

#### 2.2.3. pMT12

To conditionally express *mabR*_MS_ in *M. smegmatis*, plasmid pMR10 was digested with *Nde*I and *EcoR*I, and the fragment containing *MSMEG_4324* was cloned under the control of P*_myc1_tetO* plasmid pCK3, previously digested with the same enzymes, yielding pMT12.

#### 2.2.4. pMT13

P*_smyc_-tetR* was digested with *Sac*II and *EcoR*I from pFRA42B and cloned into pMP349 [29], previously digested with the same enzymes, yielding pMT13.

#### 2.2.5. pMR104

The fragment containing the promoter region P*fasII*_MT_ of 807 bp used for transcriptional fusion to *lacZ* was generated by PCR amplification from *M. tuberculosis* H37Rv genomic DNA with primers N800 fas2 (5′-AGTACTGCGCTGCGCTGACCGACGTG-3′) and ScaIC-Fas2TBprom (5′-AGTACTCGAACCCTGTCCGGGTGCGAGCA-3′); the *Sca*I sites are underlined. The PCR-amplifified fragment was digested with *Sca*I and ligated into the *Sca*I site of the integrative vector pSM128 [30], generating the mycobacterial reporter plasmid pMR104. The pSM128 vector carries a *cII–lacZ* fusion, the mycobacteriophage L5 integrase gene and its attachment site, and a streptomycin/spectinomycin resistance cassette. DNA sequence of the cloned fragment was determined by automated sequencing to both confifirm the sequence of the amplifified product and check the in-frame transcriptional fusion of the insert to the promoterless *lacZ* gene.

#### 2.2.6. pMT4

The fragment containing the promoter region of mutated P*fasII*_MT_ of 807 bp (800Mut2) was generated by a PCR-based method with two rounds of PCR. In the first round, two fragments were generated using two different plasmids as templates: one fragment was amplified from pMR104 using as primers N-800fas2 (5′-AGTACTGCGCTGCGCTGACCGACGTG-3′) and C-mut2frag800 (5′-TGACACGGCATTGCTGTCGATGCTT-3′); the second fragment was amplified from pMut2box [17], a pBluescript derivative that contains the 272 bp P*fasII* region with the first and second inverted repeats (IRs) replaced by a random sequence, using as primers N-fas2MtProm (5′-AAGCATCGACAGCAATGCCGTGTCA-3′) and C-fas2MtProm (5′-CGAACCCTGTCCGGGTGCGAGCAAC-3′). The generated fragments were then mixed for hybridization with their complementary sequences and after 10 cycles of amplification, external primers N-800fas2 and ScaIC-fas2TBProm (5′-AGTACTCGAACCCTGTCCGGGTGCGAGCA-3′) were added to the reaction mixture for the completion of the second round of PCR. The final fragment was digested with *Sca*I and ligated into the *Sca*I site of the integrative promoter-probe vector pSM128 [30], generating the mycobacterial reporter plasmid pMT4. The DNA sequence of the cloned fragment was determined by automated sequencing to confifirm the identity of the amplifified product and check the in-frame transcriptional fusion of the insert to the promoterless *lacZ* gene.

### 2.3. RNA extraction

RNA was extracted from cultures of the corresponding strains. Each cell pellet was resuspended in 1 ml of Quick zol (Kalium Technologies) and disrupted using Bioruptor^™^-UCD200 (Diagenode). The cell suspension was mixed with an equal volume of 100% ethanol and the RNA extraction was performed using Direct zol RNA MiniPrep (Zymo Research).

### 2.4. Reverse transcription and real-time qRT-PCR assay

Reverse transcription reaction was carried out using SuperScript III Reverse Transcriptase (Invitrogen) and random primers. The second-strand cDNA generated was used in qRT-PCR with green fluorochrome as the indicator dye (qPCR master mix, Biodynamics). The expression of *mabR*, *fabD*, *acpM*, *kasA*, *kasB*, *inhA*, *hadB*, *fabH*, *fas*, *acpS*, *fasR* was quantified after normalization of RNA levels to the expression of the *sigA* gene. All the primers were designed using Primer3 Plus software and their sequences are reported in the electronic supplementary material, table S3. qPCR cycling conditions were as follows: 95°C for 2 min followed by 40 cycles of 95°C for 15 s, 58°C for 15 s and 68°C for 20 s. qPCR data are presented as fold difference of expression in *mabR* cKD grown with ATc 2.5 ng ml^−1^ over that in the isogenic strain WT-pMT13 grown with ATc 2.5 ng ml^−1^, using the P*faffl* method [31]. The results shown are the means of three independent experiments.

### 2.5. Lipid analysis

#### 2.5.1. Fatty acid and mycolic acid analysis using thin-layer chromatography

FA and MA biosynthesis were analysed by incorporation of [1-^14^C] acetate. *Mycobacterium smegmatis mabR* cKD and WT-pMT13 were grown with ATc 2.5 and 25 ng ml^−1^ and incubated at 37°C with gentle shaking (180 r.p.m.). At T1, aliquots of 5 ml were radiolabelled with 1 µCi ml^−1^ of [1-^14^C] acetate (50.5 mCi mmol^−1^; PerkinElmer) for 1 h at 37°C. Cells were then harvested by centrifugation, washed with phosphate buffer 0.1 M (pH 7.6) and stored at −80°C. FA methyl esters (FAMEs) and MA methyl esters (MAMEs) were obtained after treatment of the radiolabelled cell pellets containing the same number of cells with aqueous tetrabutyl ammonium hydroxide followed by esterification with methyl iodide and extraction with dichloromethane, as previously described [32]. The resulting solution of FAMEs and MAMEs was assayed for radioactivity in a Beckman liquid scintillation counter and then equal counts were subjected to thin-layer chromatography (TLC), using silica gel plates (TLC silica gel 60 F_254_, Merck) and hexane : ethyl acetate (9 : 1, v/v) as the developing solvent. The radioactivity of FAMEs and MAMEs on the TLC plates was visualized using laser scanner Typhoon FLA 7000 (GE Healthcare). The spots of ^14^C-labelled FAMEs and MAMEs were quantified using Gel-Pro Analyzer software and the values obtained were expressed as arbitrary units (AU).

### 2.6. Total lipid analysis

Total lipid extractions for lipidomic analysis were prepared as follows: cell pellets were washed with 50 mM ammonium acetate (pH 7.8) and transferred to 10 ml glass tubes containing 5 ml of CHCl_3_ : CH_3_OH (2 : 1, v/v). The samples were incubated overnight at 4°C with gentle agitation. After centrifugation, bacterial pellets were subjected to an additional extraction using CHCl_3_ : CH_3_OH (1 : 2, v/v) for 2 h. Organic extracts were pooled and dried under nitrogen at 4°C. Lipids were resuspended in 3 ml of CHCl_3_, washed with 3 ml of H_2_O and the organic phase was transferred to pre-weighted glass tubes, dried under nitrogen at 4°C and reweighted on a microbalance. Extracts were dissolved in CHCl_3_ : CH_3_OH (1 : 1, v/v) at 1 mg ml^−1^ and centrifuged at 3000*g* for 5 min. Lipids were analysed in an Agilent 1200 series HPLC system with a Reprospher (Dr Maish) 100 C8 column (1.8 µm × 50 mm × 2 mm). The flow rate was 0.3 ml min^−1^ in a binary gradient mode with the following elution programme: the column was equilibrated with 100% mobile phase A (CH_3_OH : H_2_O (99 : 1, v/v), containing 0.05 mM AcNH_4_). Each sample (2 µl), containing 13 µg of total lipids in CHCl_3_, was injected and the same elution conditions continued for 1 min, followed by a 12 min gradient to 100% mobile phase B (isopropanol : hexane Reprospher (Dr Maish) H_2_O (79 : 20 : 1, v/v), containing 0.05 mM AcNH_4_) and holding for 1 min at that condition. The column was equilibrated for 2 min with 100% mobile phase A before injection of the next sample. An Agilent 6500 series Q-TOF instrument with a Dual AJS ESI was used for mass analysis. Ionization gas temperature was maintained at 200°C with a 14 l min^−1^ drying gas flow, a 35 psi nebulizer pressure and 3500 V. Spectra were collected in positive and negative mode from *m/z* 115–3000 at 4 spectra s^−1^. Continuous infusion calibrants included *m/z* 121.051 and 922.010 in positive-ion mode and *m/z* 119.035 and 955.972 in negative-ion mode. CID-MS was carried out with an energy of 50 V. For the comparative analysis, the column is conditioned by four successive mock injections with solvent cycling before randomized QCs and mycobacterial samples are analysed.

### 2.7. Expression and purification of MabR_TB_ and MabR_MS_

Expression of *mabR*_TB_ and *mabR*_MS_ were carried out following IPTG induction of BL21 *E. coli* transformed with pMR8 and pMR10, respectively. His_6_-MabR_TB_ and His_6_-MabR_MS_ were then affinity-purified from BL21 lysates using Ni-NTA agarose (Qiagen) according to the manufacturer's instructions.

### 2.8. Electrophoretic mobility shift assays

Purified recombinant His_6_-MabR_TB_ was used to assess protein binding to the *fasII* promoter fragment (272 bp). The probe was generated by PCR amplification of the *fasII* promoter from *M. tuberculosis* genomic DNA with primers N-fas2MtProm (5′-AAGCATCGACAGCAATGCCGTGTCA-3′) and C-fas2MtProm (5′-CGAACCCTGTCCGGGTGCGAGCAAC-3′) end-labelled with [γ-^32^P]-ATP (3000 Ci mmol^−1^) using T4 polynucleotide kinase. A fixed concentration of His_6_-MabR_TB_ (1 nM) was pre-incubated with acyl-CoAs at different final concentrations prior to mixing with the ^32^P-labelled probe (10 000 c.p.m.) in a total volume of 15 µl of binding buffer (25 mM Tris–HCl (pH 8), 1 mM PMSF, 5% (v/v) glycerol, 5 mM MgCl_2_, 150 mM NaCl and 1 µg poly(dIdC)) at room temperature for 15 min. DNA–protein complexes were solved by electrophoresis on a 5% (w/v) non-denaturing polyacrylamide gel in 0.5× TBE, 5% (v/v) glycerol and run at 200 V at 4°C. The gels were visualized and digitalized with a Storm 840 scanner (Amersham).

### 2.9. Protein methods

Purified proteins were analysed by SDS–PAGE [33]. Coomassie Brilliant Blue was used to stain protein bands. Protein contents in bacterial lysates were determined using the Quant-iT™ Protein Assay Kit and Qubit^®^ fluorometer (Invitrogen). Proteins were electroblotted onto nitrocellulose blotting membranes (Amersham Protran Premium 0.45 NC, GE Healthcare) for western blot analysis. MabR_MS_ was detected using 1 : 100 dilution of polyclonal anti-MabR_MS_ serum and antigenic polypeptides were visualized using a horseradish-peroxidase-conjugated secondary antibody. To identify RpoB_MS_, the membrane was probed with a 1 : 20 000 dilution of polyclonal anti-RpoB_TB_ serum, and antigenic polypeptides were visualized using an alkaline-phosphatase-conjugated secondary antibody. Antiserum against His_6_-MabR_MS_ was elicited in rabbits and anti-RpoB in mice.

### 2.10. β-Galactosidase assays

For the FA supplementation assay, saturated cultures of *M. smegmatis* MS26-P*fasII*800 grown in 7H9 with Kan and Str were diluted in the same medium to an initial OD_600_ of 0.01 and incubated at 37°C. Once the culture reached OD_600_ = 0.2, it was divided into three equal fractions and supplemented with FAs at a final concentration of 0.15 mM. Following 6.5 h of incubation, 10 ml of each culture was pelleted, washed and finally resuspended in Z buffer [34]. For INH treatment studies, saturated cultures of *M. smegmatis* MS-P*fasII*800 and MS-P*fasII*800Mut2 were also diluted to an initial OD_600_ = 0.01 and incubated at 37°C. Once the cultures reached OD_600_ = 1, each of them was divided into two equal fractions; one was treated with INH 100 μg ml^−1^ and the other served as an untreated control. After 90 min of treatment, cells were collected, washed and resuspended in Z buffer. Subsequently, the collected cells were disrupted by using Bioruptor™ -UCD200 (Diagenode), and the cell wall fractions were pelleted by centrifugation at 23 000*g* (30 min, 4°C). The resulting supernatants were quantified with the Quant-iT™ Protein Assay Kit according to the manufacturer's instructions (Invitrogen) and assayed for β-galactosidase activity [34]. The data were recorded in triplicates and levels of activity are expressed as nmol *o*-nitrophenyl-β-galactoside (ONPG) per minute per milligram of protein, and values are the means of the results ± standard deviations. Controls of background activity were made using the promoterless *lacZ* fusion (pSM128).

## 3. Results

### 3.1. Construction and growth characterization of a tight *mabR* conditional mutant in *Mycobacterium smegmatis*

**Figure 1.**
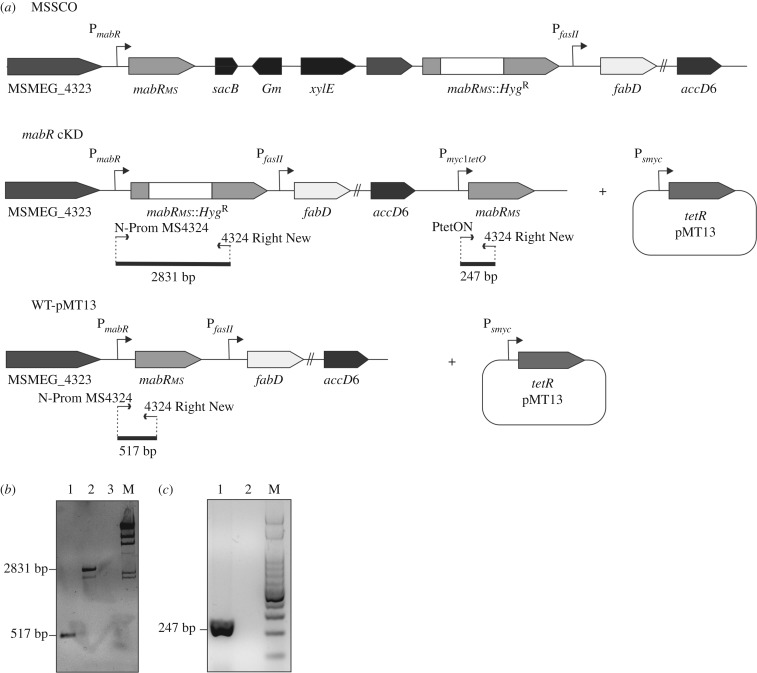
(*a*) Schematic representation of the *fasII* loci of three strains derived from *M. smegmatis* mc^2^155: MSSCO, *mabR* cKD and WT-pMT13. (*b*,*c*) The correct genetic organization of the *M. smegmatis* conditional mutant *mabR* cKD was verified by PCR. (*b*) The legitimate intrachromosomal allelic exchange in MSSCO, which leads to the conditional mutant *mabR* cKD, was verified by PCR using the oligonucleotides N-Prom MS4324 and 4324 Right New. The gel shows the amplification of genomic DNA of *mabR* cKD (lane 1) and WT-pMT13 (lane 2). Lane 3: negative control; M: molecular weight marker λ *Hind*III (PB-L Productos Bio-Lógicos). (*c*) The pair of oligonucleotides PtetON and 4324 Right New were specifically designed for the amplification of P*_myc1_tetO–mabR_MS_* fusion. Lane 1 shows the amplification product obtained from genomic DNA of *mabR* cKD and lane 2 from genomic DNA of WT-pMT13. M: molecular weight marker ladder 100 bp (PB-L Productos Bio-Lógicos).

**Figure 2.**
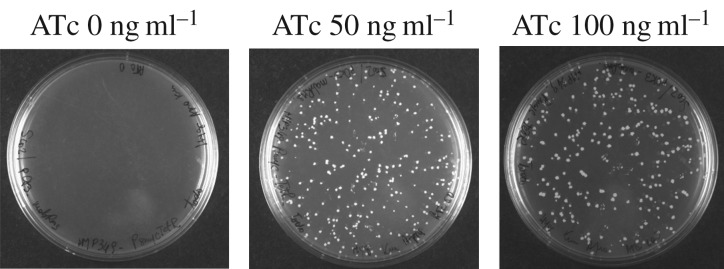
Selection of *mabR* cKD conditional mutant. The desired *mabR/mabR::hyg* allelic replacement (Hyg^r^, Kan^r^, Am^r^, Suc^r^ and XylE^−^) was only obtained in the presence of ATc. No transformants were obtained when plated on LB-Hyg-Kan-Am-Suc without ATc.

**Figure 3.**
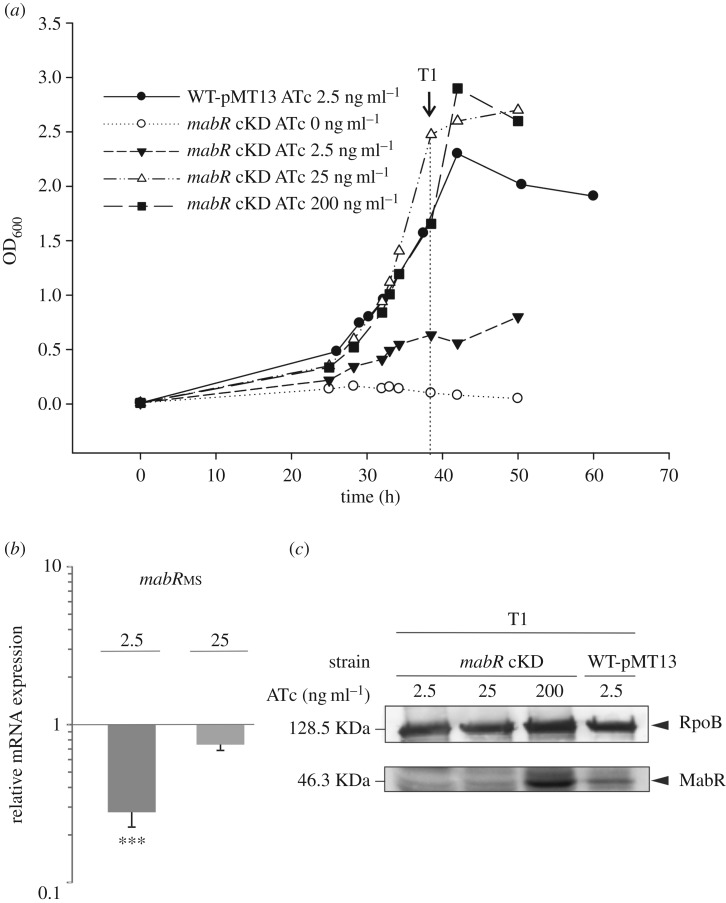
(*a*) Growth curves of WT-pMT13 and *mabR* cKD strains incubated at 37°C in the absence or presence of ATc 2.5, 25 and 200 ng ml^−1^. Saturated cultures of WT-pMT13 and *mabR* cKD grown at 37°C in the presence of ATc 50 ng ml^−1^ were washed twice with fresh 7H9 medium, then diluted in the same medium to an OD_600 nm_ of 0.01 and further incubated at 37°C. Samples were taken at T1 for analysis. (*b*) Changes in the relative amounts of *mabR* mRNA measured by quantitative RT-PCR. Values represent the mean difference of three independent experiments between *mabR* cKD and WT-pMT13 grown with ATc 2.5 or 25 ng ml^−1^ and were normalized using *sigA* as an invariant transcript. Samples for RNA extraction were collected at T1. Error bars show s.e.m. from biological replicates. Statistical significance was determined by two-tailed unpaired *t*-test, ***p < 0.001 for mabR cKD grown with ATc 2.5 ng/ml compared with WT-pMT-13. (*c*) Western blot analysis of total crude lysates from *mabR* cKD grown with ATc 2.5, 25 and 200 ng ml^−1^. As control strain we loaded WT-pMT13 grown with ATc 2.5 ng ml^−1^. Samples were collected at T1. Detection was performed using anti-MabR_MS_ antibodies raised in rabbit and anti-RpoB was used as a loading control.

To unequivocally define the physiological role of MabR in the MA biosynthesis pathway and more broadly in the lipid homeostasis of mycobacteria, we constructed a new *mabR* conditional mutant in *M. smegmatis*. Previous *in vivo* studies carried out in a *M. smegmatis* strain where MabR_MT_ was overexpressed in a multicopy plasmid from the *Pami* promoter suggested that MabR was a transcriptional repressor of the *fasII* operon [17]. However, there are several indications that studies performed with transcriptional regulators under conditions of high overexpression could result in non-physiological responses, especially in cases where the transcription factor is present in very low levels, as is the case of MabR [4]. Therefore, we decided to revisit the regulatory role of MabR on the transcriptional regulation of the *fasII* operon genes, and thus its impact on lipid metabolism, in conditions where MabR levels were below its physiological concentrations. As it is impossible to generate a *mabR* knockout mutant due to the essentiality of this gene for *M. smegmatis* viability [17], a tight conditional mutant was constructed using a TetR/*tetO* expression regulation system. The single crossover event MSSCO, carrying both an intact and a disrupted copy of *mabR* (*mabR::hyg*), was previously constructed by Salzman *et al.* [17]. To obtain the *mabR/mabR::hyg* allelic replacement, a merodiploid strain was obtained by stably integrating a second copy of *mabR_MS_* at the L5 phage attachment site *attB* under the control of TetR. For this, we introduced two plasmids in MSSCO: pMT12, an integrative plasmid containing a wild-type copy of *mabR*_MS_ under the control of *P_myc1_tetO*; and pMT13, a replicative plasmid that constitutively expresses the TetR repressor from *Psmyc* (figure 1*a* and Materials and methods). The transformants were selected on Hyg-Kan-Am-Suc plates in the absence or presence of different concentrations of ATc, the chemical inducer for this gene regulation system. No transformants were obtained when plated in the absence of ATc, confirming the essentiality of *mabR* for *M. smegmatis* viability (figure 2). However, transformants were obtained in plates containing ATc 50 ng ml^−1^, suggesting the requirement of *mabR* expression for cell growth. When these transformants were sprayed with catechol, the colonies were white, indicating a successful *mabR/mabR::hyg* intrachromosomal allelic exchange. All these clones should have the endogenous copy of *mabR* interrupted with a Hyg resistance cassette and an extra copy of *mabR* integrated ectopically into the genome whose expression relies on the presence of ATc (figure 1). The predicted genotype was confirmed by genomic PCR (figure 1*b*,*c*). The resulting conditional mutant strain, named *mabR* cKD, and the WT-pMT13 were cultivated in 7H9 with or without ATc and bacterial growth evaluated by measuring OD_600_ at different time points. The mutant strain exhibited a growth curve very similar to the wild-type strain at concentrations of ATc ≥ 25 ng ml^−1^; however, in the absence of ATc, no growth was observed (figure 3*a*). From these experiments, we can conclude that there is a threshold level of MabR above which the bacteria grow normally (figure 3*a*,*c*). The parental strain WT-pMT13, harbouring the plasmid that expresses TetR constitutively, was unaffected by the presence of ATc up to 200 ng ml^−1^ (data not shown).

### 3.2. MabR is a transcriptional activator of the *fasII* operon genes in mycobacteria

To study the impact of *mabR* depletion in lipid metabolism, we set up to analyse in detail those cultures grown in the presence of ATc 2.5 ng ml^−1^, where the conditional mutant *mabR* cKD exhibited lower levels of MabR expression in comparison with the WT-pMT13 strain (figure 3*b*,*c*).

**Figure 4.**
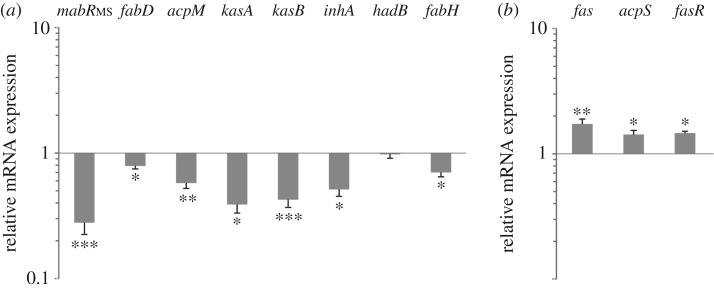
The reduced mRNA expression of *mabR* leads to downregulation of genes required for MA biosynthesis (*a*) and upregulation of *fas-acpS* operon (*b*). (*a* and *b*) The relative expression profile of *mabR*, *fabD*, *acpM*, *kasA*, *kasB*, *inhA*, *hadB*, *fabH*, *fas*, *acpS and fasR* was determined by qRT-PCR at T1. The cycle threshold (CT) value of each gene was normalized by using the housekeeping gene *sigA*. Values represent the mean difference between *mabR* cKD and WT-pMT13 grown with ATc 2.5 ng ml^−1^, performed in triplicate. Error bars show s.e.m. from biological replicates. Statistical significance was determined by two-tailed unpaired *t*-test, *p < 0.05, **p < 0.01, ***p < 0.001.

To characterize the regulatory role of MabR on the expression of the genes involved in MA biosynthesis, we first analysed the expression of the *fasII* operon genes in the conditional mutant *mabR* cKD presenting reduced MabR levels, and compared it with the expression profile of WT-pMT13. The relative amounts of *fabD*, *acpM*, *kasA* and *kasB* mRNAs were measured by quantitative RT-PCR at T1 of the growth curves of the conditional mutant *mabR* cKD and the WT-pMT13 grown in the presence of ATc 2.5 ng ml^−1^. As shown in figure 4*a*, the transcription of all the *fasII* operon genes is turned down when the cells have reduced MabR expression levels. These results support the idea of MabR being a transcriptional activator, and not a repressor, of the *fasII* operon. Moreover, the expression of other genes related to MA biosynthesis, but located in different genome loci, was also shown to be downregulated, as depicted for *inhA* and *fabH* in figure 4*a*. On the other hand, the expression of the *fas-acpS* operon (encoding for the multidomain FAS I and the phosphopantetheinyl transferase AcpS, respectively) was upregulated in conditions of reduced MabR levels (figure 4*b*). Overall, these studies confirm that MabR is a transcriptional activator of the *fasII* operon and that suboptimal levels of this regulator also affect the expression of other genes that form part of the FAS I and FAS II systems.

### 3.3. Turn-down expression of *mabR* results in inhibition of mycolic acid biosynthesis

**Figure 5.**
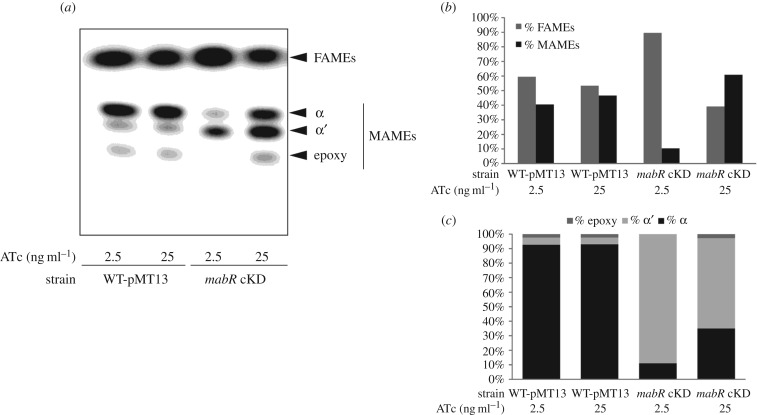
*De novo* FA and MA biosynthesis. (*a*) Cells from *mabR* cKD and WT-pMT13 cultures, grown in the presence of ATc 2.5 and 25 ng ml^−1^, were labelled with [^14^C] acetate at T1 for 1 h at 37°C. After organic extraction, equal counts (20 000 c.p.m.) of ^14^C-labelled methyl esters of MAs (MAMEs) and FAs (FAMEs) were spotted on a TLC plate. Separation was performed using hexane : ethyl acetate (9 : 1 v/v) as the solvent system. (*b* and *c*) Quantification of the radiolabelling intensity is shown. (*b*) Relative distribution of FAMEs and MAMEs. (*c*) Relative distribution among the three species of MAMEs.

To study the impact of sub-physiological levels of MabR in the *de novo* synthesis of FA and MA, [^14^C]-acetate labelling experiments were carried out with *mabR* cKD and WT-pMT13 strains. Both strains were grown with ATc 2.5 and 25 ng ml^−1^, and their lipids were extracted and analysed by radio-TLC. As shown in figure 5*a*, the *de novo* synthesis of MA in *mabR* cKD was strongly inhibited when the cells were grown in the presence of ATc 2.5 ng ml^−1^, where the levels of MabR were much lower compared with the physiological amounts present in the WT-pMT13 strain. However, FA biosynthesis was not affected, even when MA biosynthesis was extremely compromised, indicating that a normal functioning of the FAS I system is not sufficient for the survival of the cells due to the essential role of MAs in the architecture of the cell wall. On the other hand, although total amount of the *de novo* MA biosynthesis was not affected in the *mabR* cKD strain grown in the presence of ATc 25 ng ml^−1^ (figure 5*a*,*b*), there were clear differences between the relative abundance of the three classes of MAMEs, which could correlate with the slightly lower levels of MabR detected under these conditions (figures 3*b* and 5*c*). The relative percentages for α-, α′- and epoxy-mycolates from the WT-pMT13 strain grown in the presence of ATc 2.5 and 25 ng ml^−1^ were identical, 93%, 4.5% and 2.5%, respectively (figure 5*c*), while the profiles obtained for the *mabR* cKD strain grown with ATc 25 ng ml^−1^ were 35%, 62% and 3%, respectively, showing a significant increase in the abundance of α′-mycolates (62%) with a compensatory reduction of α-mycolates (figure 5). In addition to this, within the low amount of MA synthesized *de novo* in *mabR* cKD grown in the presence of ATc 2.5 ng ml^−1^, there is a prevalence of α′-mycolates (89%) compared with the other MA species (figure 5*c*).

### 3.4. Sub-physiological concentrations of MabR have a global impact on lipid composition in *Mycobacterium smegmatis*

**Figure 6.**
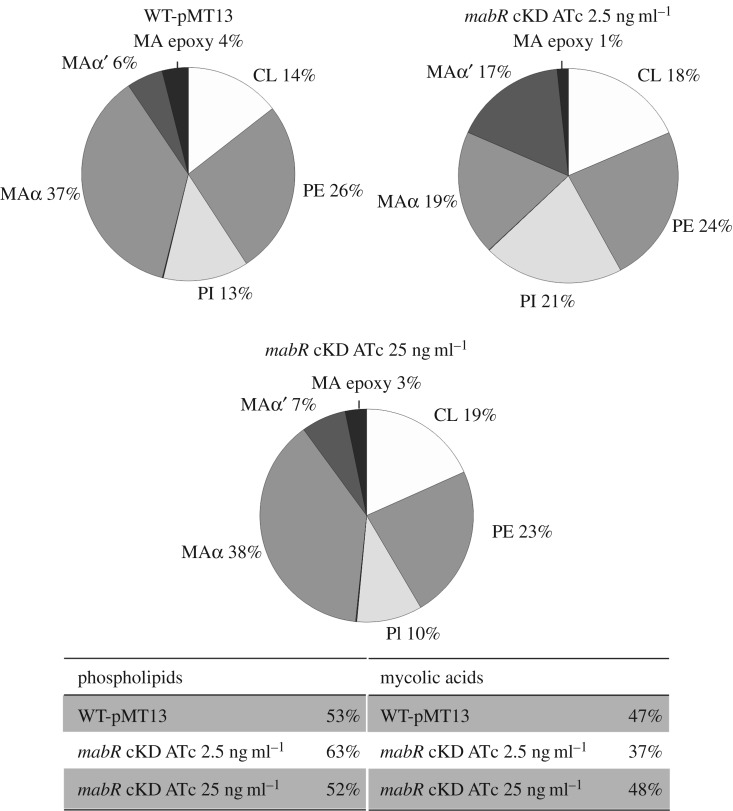
LC–MS analyses of the PL and MAs present in the *mabR* cKD mutant and in the isogenic strain WT-pMT13. The values indicated represent the relative abundance of the MS signals corresponding to the different lipid classes found in the samples. Results are the means of three independent experiments. MA, mycolic acids; CL, cardiolipin; PE, phosphatidylethanolamine; PI, phosphatidylinositol.

Based on the results shown in figure 5, we decided to expand our studies and determine the impact of sub-physiological levels of MabR on the global lipid profile. For this we performed comparative lipidomic analyses between the conditional *mabR* cKD mutant and its parental strain WT-pMT13. Briefly, cell-associated lipids were extracted from the conditional *mabR* cKD mutant grown in the presence of ATc 2.5 and 25 ng ml^−1^, and the total lipid content was analysed by high-performance liquid chromatography–mass spectrometry (HPLC-MS). As a control, we extracted and analysed lipids from the parental control strain WT-pMT13 grown in the presence of ATc 2.5 ng ml^−1^. Figure 6 shows the results obtained for the lipid analyses carried out in negative-ion mode. The most significant differences were found in the content and relative composition of MAs, as was previously observed in the radio-TLC analyses, where the mutant grown in the presence of ATc 2.5 ng ml^−1^ showed a significant decrease in MAs and a higher proportion of α′ mycolates compared with the control strain. Notably, the distribution of various MAs was shifted to shorter forms in the mutant with reduced levels of MabR (electronic supplementary material, figure S1*a*). Furthermore, tandem mass analysis (MS–MS) of the individual types of mycolates showed that α-branches of the mycolates from WT-pMT13 and *mabR* cKD strains grown in the presence of ATc 2.5 ng ml^−1^ contained mainly 24 carbons, indicating that the length difference of these molecules corresponded to the mero-chains (electronic supplementary material, figure S1*b*). On the other hand, the relative composition of PL showed a more biased effect towards phosphatidylinositol (PI), which was significantly increased in the presence of sub-physiological levels of MabR (figure 6).

Complex lipids bearing MA moieties, such as glycerol monomycolates (GroMM), monomeromycolyl diacylglycerol (MMDAG) or trehalose monomycolates (TMM), were analysed in the positive-ion mode, and they were also found in lower amounts when the mutant strain had lower levels of MabR (table 1). This result reflects the impact that this transcriptional regulator has in probably all MA-containing molecules. Furthermore, the relative content of other neutral lipids, such as DAG (diacylglycerol) and TAG, was not significantly affected, although the average size of TAG was shortened in the conditional mutant *mabR* cKD grown in the presence of ATc 2.5 ng ml^−1^ (electronic supplementary material, figure S2*a*,*b*).

**Table 1.** Neutral lipid analysis.

strain	TAG (%)	MMDAG (%)	DAG (%)	TMM (%)	GroMM (%)
WT-pMT13	95.7	3.02	0.61	0.47	0.17
*mabR* cKD ATc 2.5 ng ml^−1^	98.6	0.71	0.54	0.10	0.05
*mabR* cKD ATc 25 ng ml^−1^	96.3	2.82	0.46	0.30	0.14

### 3.5. Upregulation of *fas II* operon expression as a response to isoniazid is MabR-dependent

**Figure 7.**
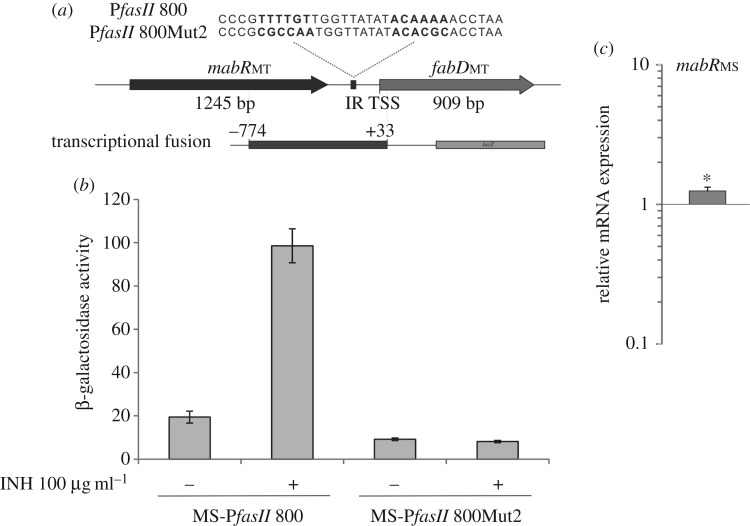
The *fasII* operon activation upon INH treatment is MabR-dependent. (*a*) Schematic of the intergenic space between *mabR*_MT_ and *fabD*_MT_ from *M. tuberculosis*. The inset shows the highly conserved palindromic inverted repeats (IRs) (indicated in bold) present in the sequence of P*fasII* 800 and a variant of this DNA motif where the IRs were replaced with random sequences (P*fasII* 800 Mut2). The transcriptional fusion used for the assays is depicted, showing its boundaries. (*b*) Intracellular β-galactosidase activity of MS-P*fasII* 800 and MS-P*fasII* 800Mut2 strains with and without INH treatment. Levels of activity are shown as nmol ONPG per min per mg of protein and are the means of the results of three independent experiments ± s.d. (*n* = 3). (*c*) Changes in the relative amounts of *mabR* mRNA measured by quantitative RT-PCR. Values represent the mean difference between *M. smegmatis* mc^2^155 treated for 90 min with INH and the control without treatment, and are normalized using *sigA* as an invariant transcript. Statistical significance was determined by the two-tailed unpaired *t*-test, *p < 0.05.

Mdluli *et al.* [35] showed that INH treatment of *M. tuberculosis* inhibits MA synthesis and is accompanied by a marked upregulation of both AcpM and KasA. Later, transcriptomic studies conducted by several laboratories to explore the genome-wide transcriptional response of *M. tuberculosis* to different drugs demonstrated that INH and ETH, both potent inhibitors of InhA, markedly induced the transcription of the *fasII* operon genes [36,37]. More recently, Salzman *et al.* [17] showed that MabR is a transcriptional regulator that modulates the expression of the *fasII* operon. However, it is still an open question whether or not the transcriptional activation of the *fasII* operon genes upon INH treatment is mediated by MabR. Therefore, to investigate whether MabR is involved in this regulation, we constructed two transcriptional fusions to the reporter gene *lacZ*: P*fasII*800_MT_::*lacZ* and P*fasII*800_MT_Mut2::*lacZ.* The first transcriptional fusion comprises 774 bp upstream of the TSS of *fabD* and corresponds to the wild-type sequence of *PfasII*_MT_, which includes the MabR recognition sequence 5′-TTTTGT(N)_9_ACAAAA-3′; the second transcriptional fusion P*fasII*800_MT_Mut2::*lacZ* corresponds to a variant of this promoter region where the palindromic IRs were replaced by the random sequences 5′-CGCCAA(N)_9_ACACGC-3′ (figure 7*a*). Using gel shift assays, we had previously demonstrated that the mutated IRs were unable to bind MabR [17]. Once the two transcriptional fusions were constructed, they were cloned into the integrative plasmid pSM128 and independently transformed into *M. smegmatis* mc^2^155, generating two strains (MS-P*fasII*800 and MS-P*fasII*800Mut2), which were used for transcriptional activity studies by assaying β-galactosidase activity in their cell-free extracts. As showed in figure 7*b*, after the treatment of the cultures of both strains with INH 100 μg ml^−1^ for 90 min, the β-galactosidase activity present in MS-P*fasII*800 extracts showed a five-fold increase compared with the activity levels found in the non-treated cells, while we did not find variation for MS-P*fasII*800Mut2 treated or not with INH. This result confirmed that MabR binding sites, and therefore MabR, are required for the transcriptional activation of the *fasII* operon upon INH treatment. Moreover, this experiment also corroborated the activating role of MabR in the transcriptional regulation of the *fasII* operon.

To determine if the INH-mediated induction of P*fasII* could be correlated with a change in the expression level of *mabR*, total RNA was purified from *M. smegmatis* cultures treated or not with INH and analysed by quantitative RT-PCR. As shown in figure 7*c*, *mabR* expression showed a modest increase (about 30%) after INH treatment.

### 3.6. Long-chain acyl-CoAs increase the binding affinity of MabR for the *fasII* promoter

**Figure 8.**
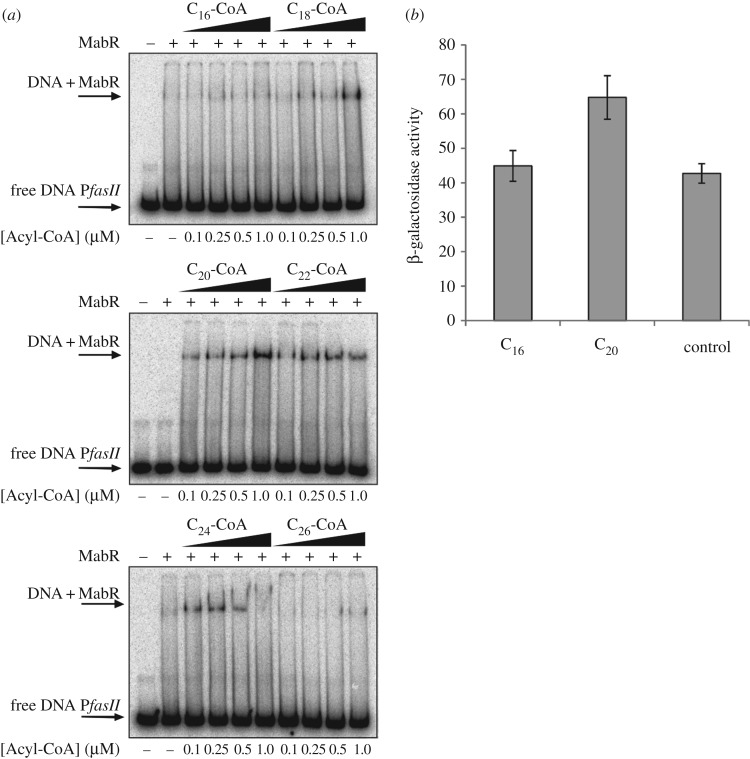
(*a*) Binding of MabR to *fasII* promoter region is enhanced by long-chain fatty acyl-CoAs. Gel shift assays were performed by incubating a ^32^P-labelled 272 bp P*fasII* probe with a fixed amount of MabR_MT_ in the presence of increasing levels of medium- and long-chain acyl-CoAs (0.1–1 µM). (*b*) Long-chain FAs enhance the activation of *fasII* operon *in vivo* by increasing the binding affinity of MabR to the *fasII* promoter. Intracellular β-galactosidase activity of the strain MS26-P*fasII* 800 grown in 7H9 medium in the presence of different FAs at final concentration of 0.15 mM. The control was performed growing the strain in the same medium without supplementing with FAs. Samples of each culture were removed 6.5 h post-induction to assay β-galactosidase specific activity. Levels of activity are shown as nmol ONPG per min per mg of protein and are means of the results of three independent experiments ± s.d. (*n* = 3).

The final products of the bacterial FAS II system are well-established signal molecules that control FAS II activity by binding to the enzymes involved in the biosynthetic pathways [38,39] or by modulating the affinity of the transcriptional regulators to their target DNA [40]. Thus, in order to identify the effector molecule(s) recognized by MabR and that modulate its transcriptional activity, we surveyed a set of compounds related with mycobacteria lipid metabolism by analysing their ability to modulate the binding of MabR_MT_ to P*fasII*_MT_ (a 272 bp probe which consists of 239 bp upstream and 33 bp downstream of the TSS of *fabD*, including the MabR binding site). These compounds included palmitoyl-CoA (C_16_-CoA), stearoyl-coA (C_18_-CoA), arachidoyl-CoA (C_20_-CoA), behenoyl-CoA (C_22_-CoA), lignoceroyl-CoA (C_24_-CoA) and hexacosanoyl-CoA (C_26_-CoA). In these experiments, the reaction mixture contained, in addition to the test compounds added at different final concentrations (0.1, 0.25, 0.5 and 1 µM), ^32^P-labelled P*fasII*_MT_ DNA and 1 nM His_6_-MabR_MT_. To appreciate even slight differences in the binding affinity of MabR to its DNA probe upon the addition of the test molecules, the concentration of His_6_-MabR_MT_ used for these gel shift assays was below the one that allows a complete shift of the probe P*fasII*_MT_. As shown in figure 8, the addition of C_16_-CoA had no effect on the binding affinity of MabR to the probe, while the presence of C_18_-CoA increased the affinity of the regulator towards P*fasII*_MT_ when assayed at a final concentration of 1 µM (figure 8*a*). When the reaction mixture was supplemented with C_20_-CoA and C_22_-CoA, the binding affinity was enhanced throughout the range of concentrations studied. Remarkably, although the addition of C_24_-CoA stabilized the interaction between MabR and the DNA probe, higher concentrations of this acyl-CoA (0.5 and 1 µM) provoked its dissociation or the inhibition of the binding. This effect could be considered as the result of protein denaturation by the detergent action of the long-chain acyl-CoA at higher concentrations, reducing the levels of native MabR available for the formation of the protein–DNA complex. C_26_-CoA did not show an effect on promoting MabR–DNA interaction. This result could have been obtained either because C_26_-CoA is not a ligand recognized by MabR or because it could be denaturing the regulatory protein. To tackle this question, MabR was pre-incubated with C_26_-CoA at a final concentration of 0.5 µM for 5 min and then C_20_-CoA was added to the final concentration of 0.5 or 1 µM, together with a ^32^P-labelled probe. In this reaction, no MabR–DNA complex was formed, thus suggesting a denaturing effect of C_26_-CoA towards MabR, which is then not available to bind the P*fasII* probe, even when C_20_-CoA was present in the reaction mixture to promote this interaction (electronic supplementary material, figure S3).

Altogether, our studies demonstrate that long-chain acyl-CoAs (longer than C_18_) could be the signal molecules that modulate the binding between MabR and the P*fasII* promoter, at least *in vitro.*

### 3.7. *In vivo* effect of arachidic acid in the modulation of *fasII* operon gene expression

The fact that MabR is a transcriptional activator of the *fasII* operon and long-chain acyl-CoAs can modulate the binding between MabR and P*fasII* promoter *in vitro* strongly suggests that these activated long-chain FAs are the ligands that modulate the expression profile of *fasII* operon via MabR and help in coordinating the activity of the two FAS systems to keep lipid homeostasis tightly regulated. Therefore, in order to study the ability of different chain-length acyl-CoAs to modulate the activity of P*fasII* promoter *in vivo*, we assayed the response of this promoter in a strain of *M. smegmatis* harbouring the P*fasII*800_MT_::*lacZ* transcriptional fusion when supplementing the media with palmitic acid (C_16_) or arachidic acid (C_20_), which are converted into their corresponding CoA derivatives once within the cell. The final strain also carried the replicative plasmid pMR26, which contains a copy of *mabR*_MT_ under the leaky P*ami* promoter. In this experiment, cells were grown in 7H9 medium without acetamide to allow the presence of basal levels of MabR_MT_. Once the cell culture reached OD_600_ = 0.2–0.3, it was divided into three equal fractions, two of them were supplemented with C_16_ or C_20_ to a final concentration of 0.15 mM, and a control culture was left without the addition of FAs. Samples were taken 6.5 h after supplementing the cultures with the specific FAs for β-galactosidase analysis. As shown in figure 8*b*, the presence of palmitic acid in the growth medium has no enhancing effect on the transcriptional activity of the *lacZ* gene when compared with the control culture, while arachidic acid led to increased levels of β-galactosidase activity, suggesting an activation of the *fasII* operon transcription once MabR binds to C_20_-CoA. This result coincides with the effect observed in the *in vitro* experiments shown in figure 8*a*, where C_16_-CoA had no effect on the *in vitro* MabR–P*fasII* interaction while C_20_-CoA enhanced this binding. All these results point to long-chain acyl-CoAs being the metabolic signals sensed by MabR to exert its role as a transcriptional activator of the *fasII* operon.

## 4. Discussion and conclusion

The *M. tuberculosis* mycomembrane is a highly efficient permeability barrier containing many antigenic determinants and is essential to cell viability and virulence. The major components of the mycomembrane are the MAs, which play a central role in the process of infection and persistence within the host. During the process of infection, the bacteria must continuously adapt to various environmental conditions by regulating its energy metabolism and cell wall composition. To finely control cell wall biosynthesis, *M. tuberculosis* employs many sophisticated regulatory strategies such as transcriptional regulation of lipid genes [17,19] or modulation of the activities of key enzymes in MA synthesis by post-translational modification [16,41–43]; this is similar to the mechanisms employed by other bacteria to control their FA metabolism, as has been well studied in *E. coli* [44–46], *Streptococcus pneumoniae* [47] and *Bacillus subtilis* [46,48]. In this way, by adjusting the levels and activities of MA biosynthetic enzymes, mycobacteria could match the demand for new membrane synthesis and extension of its complex cell wall.

Unlike the transcriptional regulators involved in lipid metabolism in other bacteria, MabR and FasR from *M. smegmatis* have been shown to be essential for cell viability [17,19], suggesting a key role of these proteins in keeping lipid homeostasis in this organism by coordinating the crosstalk between the two FAS systems.

In this work, by constructing an MabR conditional mutant regulated by TetR, we were able to downregulate the expression of MabR and study the physiological response of the cell to suboptimal levels of the transcriptional regulator. The complementing *in vitro* and *in vivo* studies, the latter carried out with the *mabR* cKD conditional mutant, allowed us to demonstrate that MabR is a transcriptional activator of the *fasII* operon. These results contradicted our previous studies, where MabR was previously described as a negative regulator of the *fasII* operon genes [17]. We now speculate that the different results obtained from these two independent sets of experiments could be explained by the differences found in the approach used in each case. In our former study, we used a multicopy plasmid from which *mabR* was overexpressed from the *P_ami_* promoter, implying that we characterized the system in conditions where MabR levels were very high in comparison with its physiological levels. In this regard, there are examples in the literature where overexpression of transcriptional regulators could lead to a non-physiological response of the regulatory protein due to the misinteraction of the apo form of the regulator with the DNA. In the aforementioned studies, we complemented the *M. smegmatis mabR* conditional mutant with a *mabR* copy of either *M. tuberculosis or M. smegmatis*; interestingly, the two complemented mutants grew similarly to the wild-type strain only in the absence of acetamide (the inducer of the *Pami* promoter), where the leakiness of the system allowed a weak expression of *mabR*. However, the colonies were of reduced size and displayed clear morphological differences with the wild-type strain in the presence of acetamide, and in a clear correlation with a lower expression of the *fasII* genes [17]. Therefore, we could speculate that in conditions of MabR overexpression, the regulator could serendipitously bind to the operator sequences of the P*fasII* region in the absence of its ligand, in that way inhibiting the physiological response of P*fasII* to the effector-bound form of MabR.

Interestingly, sub-physiological levels of MabR not only impacted on the expression of the *fasII* operon genes; for instance, transcription of other genes related with the FAS II system, but encoded in other genome loci, such as *inhA* (second gene of the *mabA-inhA* bicistronic operon) and *fabH*, acting at the intermediate step between FAS I and FAS II activities, was also downregulated. Analysis of the DNA sequences found upstream of the TSS of either the *mabA-inhA* operon or the *fabH* gene did not reveal consensus MabR recognition sites. Thus, this cross-regulation might occur at a higher hierarchical level where a more global regulator might act in response to a generalized stress signal generated by the inhibition of MA biosynthesis, turning down other enzymes related to this metabolism. In relation to these results, it has been recently demonstrated that starvation of *M. tuberculosis* also leads to a generalized downregulation of genes required for the synthesis (this include *fasII*, *mabA-inhA* and *hadABC* operon genes), modification and transport of mycolates [49]. As this is a highly energy-consuming process, it is physiologically relevant for the cell to slow down its synthesis during nutrient scarcity. From these studies, it is also relevant to highlight that after starvation, *mabR* expression was also turned down, in consonance with the downregulation of the MA biosynthesis genes thus supporting the activator role of this transcriptional regulator.

Interestingly, the expression of the *hadABC* genes was not modified under conditions of MabR depletion. Whether the lack of response of this operon is related to the existence of a second dehydratase (MSMEG_6754) present in *M. smegmatis* and found to have functional redundancy with HadB [50] remains to be explored. Furthermore, the transcriptional regulation of the *hadABC* operon is more complicated, because they are part of a seven-gene operon together with another four genes involved in translation, and at least in *M. tuberculosis* they all respond to the alarmone (p)ppGpp which leads to dramatic reprogramming of cell transcription [49]. Therefore, the expected coordination of the expression of the complete set of FAS II genes is far more complex and denotes an eventual interplay between different regulatory pathways.

The opposite response between the expression of *fas* and *fabH* observed in our experiments was also found during growth arrest [51]. It was proposed that this uncoupling between FAS I and FAS II systems would allow the rerouting of the carbon flux towards the synthesis of storage and stress-protective compounds, such as TAG and glutamate, with the possibility of returning the meromycolate intermediate products for the synthesis of alternative FAs. The downregulation of MabR and, therefore, the MA biosynthesis genes might lead to a similar situation where the acyl-CoAs produced by FAS I might be funnelled into the synthesis of TAG.

In the condition where the levels of MabR were below its physiological concentration, and the transcription of most of the genes involved in the FAS II system was downregulated, we found that MA biosynthesis was strongly impaired and its relative composition significantly modified, with a high prevalence of α′-MAs. It has been suggested that α′-mycolates are the precursor of α-mycolates, and KasA has been postulated to be the key enzyme responsible for elongating α′-MAs to α-MAs [52]. In a *M. smegmatis* KasA-overexpressing strain, α′-mycolates decrease with a concomitant increase of α-mycolates [13,52], showing the ability of KasA to elongate the shorter α′-chains into full-length meromycolates. It has also been demonstrated that the depletion of KasA or inhibition of its β-ketoacyl-ACP synthase activity with TLM was accompanied by an accumulation of α′-mycolates [53,54]. Thus, our results are in agreement with previous studies because upon MabR depletion there is a reduction of KasA, which results in the accumulation of α′-MAs.

In regard to the PL composition found in MabR-deprived cells, there is a relative increase in the population of PI among the PL species. Contrary to phosphatidylethanolamine, phosphatidylglycerol, phosphatidylserine and cardiolipin, which are frequently encountered in all living organisms, PI is an essential phospholipid of eukaryotic cells, but has seldom been found in prokaryotic cells. Actually, the distribution of PI in prokaryotes seems to be confined to some actinomycetes (*Mycobacterium*, *Corynebacterium*, *Nocardia*, *Micromonospora*, *Streptomyces* and *Propionibacterium*), myxobacteria, and to the genus *Treponema*. In *Mycobacterium* sp., PI and metabolically derived molecules of which PI constitutes a lipid anchor to the cell envelope, such as PI mannosides (PIMans), linear lipomannan (LM) and mature branched LM and lipoarabinomannan, are prominent and important PL/lipoglycans [55]. PI and PIMans are regarded as essential for membrane stability and thus for cell viability [56,57]. This might be the reason for PI being the PL species which suffers the most upon variations on lipid metabolism. In lipidomic studies carried out in an *M. smegmatis fas-acpS* conditional mutant, where the expression of FAS I synthase was drastically turned down, we also found that the relative amount of PI dropped significantly, while the other PL species had no alteration [13]. Preliminary proteomic analysis of conditional mutants altered in lipid metabolism lead us to propose that the PI synthase [56] could be one of the key enzymes that become regulated as a response to changes in lipid homeostasis.

Working with two different P*fasII*–*lacZ* transcriptional fusions, one containing the native MabR recognition sites and the other mutated in the palindromic IR, in a way that they were no longer recognized by MabR [17], we demonstrated that the activation of the *fasII* operon after INH treatment was MabR-dependent. Furthermore, quantitative RT-PCR experiments showed that the levels of *mabR* expression increased approximately 30% after INH treatment; therefore, we could hypothesize that either a limited increase in MabR is sufficient to induce the expression of the *fasII* genes or, the signal molecules recognized by this activator, e.g. long-chain acyl-CoAs, become accumulated after the inhibition of the FAS II system and reach a threshold to effectively bind MabR and then induce the expression of the *fasII* operon. For instance, and in analogy with other known mechanisms used by transcriptional regulators related to lipid metabolism [22,40,58], we also propose that long-chain acyl-CoAs are the ligands that bind to MabR to modify its DNA-binding properties. In fact, the regulatory activity of MabR is positively modulated by the binding of acyl-CoAs longer than C_18_. Thus, it is tempting to speculate that final products of FAS I (e.g. long-chain acyl-CoAs, precursors of the α-branch of MAs) might accumulate as a response to MA inhibition and induce at least part of the MA biosynthesis genes. In this way, the transcriptional regulator senses and responds to a variation in the signal molecules, which in fact reflect the metabolic state of the cell and adjust its lipid composition in order to adapt the bacteria to a new living condition.

In transcriptional studies carried out with FasR, the transcriptional activator of the *fas-acpS* operon of *Mycobacterium*, we demonstrated that long fatty acyl-CoAs (longer than C_18_-CoA) inhibit the binding of FasR to P*fas*, turning down the expression of the *fas-acpS* operon [19]. Therefore, these former studies and our current work lead us to propose fatty acyl-CoAs longer than C_18_ as key metabolites that are sensed by the two main transcriptional regulators charged to maintain lipid homeostasis in mycobacteria. Considering that both regulatory proteins have been shown to be essential for cell viability in *M. smegmatis*, it is tempting to speculate that they will also be essential proteins in *M. tuberculosis*, and if so, they could be considered plausible new targets for the screening of compounds that could disrupt their regular DNA-binding properties and in this way inhibit cell growth.

## Supplementary Material

Fig S1

## Supplementary Material

Fig S2

## Supplementary Material

Fig S3

## Supplementary Material

Supplementary Tables S1 - S3

## Data accessibility

Additional data have been uploaded as the electronic supplementary material.
