# Supplementary figures and images for: Role of long-chain acyl-CoAs in the regulation of mycolic acid biosynthesis in mycobacteria
Source: Open Biol. 2017 Jul 19;7(7):170087. doi: 10.1098/rsob.170087 (PMC5541348; doi:10.1098/rsob.170087)

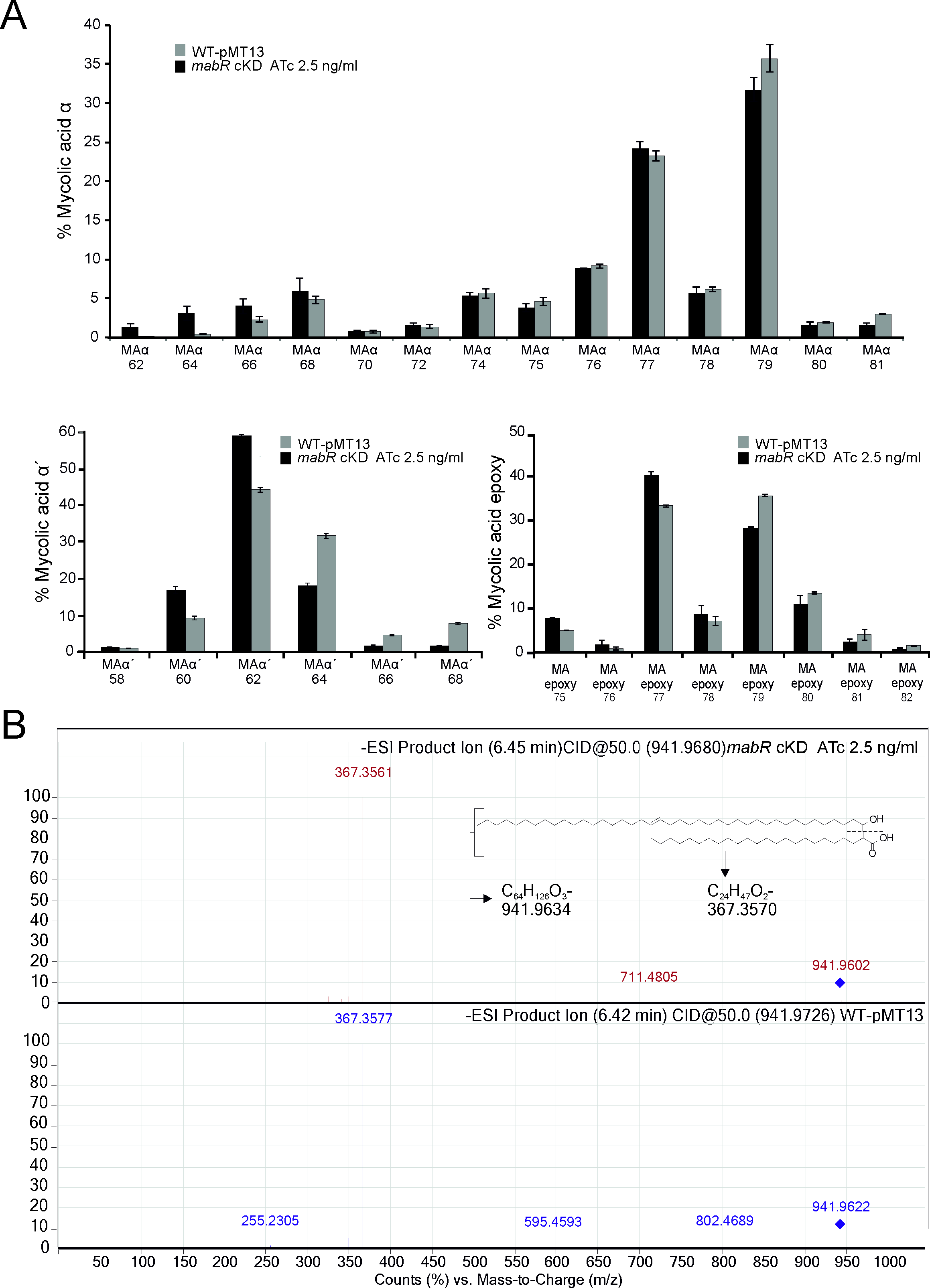

Supplement: Fig S1 [file rsob170087supp1.tif]
