# Supplementary material for: Role of long-chain acyl-CoAs in the regulation of mycolic acid biosynthesis in mycobacteria
Source: Open Biol. 2017 Jul 19;7(7):170087. doi: 10.1098/rsob.170087 (PMC5541348; doi:10.1098/rsob.170087)
Supplement: Supplementary Tables S1 - S3 [file rsob170087supp4.docx]

**SUPPORTING INFORMATION**

**S1 Table. Plasmids used in this work.**

**S2 Table. Strains used in this work.**

**S3Table. RT primers used in this study.**

**FIGURE LEGENDS TO SUPPLEMENTARY FIGURES**

**Fig S1. Detailed analysis of mycolic acid molecular species.** (A) The histograms show the distribution of the number of carbon of free α, α´ and epoxy MA in *mabR* cKD ATc 2.5 ng/ml and WT-pMT13. (B) Tandem mass spectra of the α´ MA with 64 (941.97 m/z) carbon atoms in two different samples showing the C24 α branch (367.35 m/z) signal. The inset shows where the fragmentation is produced in the MA structure.

**Fig S2. Detailed analysis of TAG molecular species.** (A) The table shows the weighted average of total number of carbon atoms per molecule within the population of TAGs of the different samples. The results are the mean of three independent experiments ± standard deviations. The P value was calculated as unpaired t test with Welch's correction between *mabR* cKD ATc 2.5 ng/ml and WT-pMT13. (B) The histogram shows the distribution of the number of carbon of TAGs in *mabR* cKD ATc 2.5 ng/ml and WT-pMT13.

**Fig S3.** **Effect of C_26_-CoA on the formation of C_20_-CoA/ His_6_-MabR_MT_ /P*fasII* complex*.*** His_6_-MabR_MT_ was preincubated during 5 min with C_26_-CoA in the presence of poly-dIdC, prior to the addition of C_20_-CoA and 5 min later, P*fasII* probe was added (last 2 lanes). The His_6_-MabR_MT_-DNA complex formation was also assayed with the individual addition of different concentrations of C_20_-CoA (lanes 3-6) or C_26_-CoA (lanes 7-10), as controls of the experiment.
